# Employer-Sponsored Health Centers Provide Access to Integrated Care via a Hybrid of Virtual and In-Person Visits

**DOI:** 10.1089/tmr.2021.0027

**Published:** 2021-11-02

**Authors:** Divya K. Madhusudhan, Sharon A. Watts, Daniel J. Lord, Fiona Ding, David C. Lawrence, Austin Sheldon, James Leonard, Dena M. Bravata

**Affiliations:** ^1^ Crossover Health, San Clemente, California, USA.; ^2^ Global Clinical Scholars Research Training Program, Harvard Medical School Postgraduate Medical Education, Cambridge, MA, USA.; ^3^ Watts Writing LLC, Akron, Ohio, USA.; ^4^ Stanford Center for Primary Care & Outcomes Research, Palo Alto, California, USA.

**Keywords:** team-based care, telemedicine, telehealth, virtual primary care, physical medicine, behavioral health

## Abstract

**Background:** Since the explosion of telemedicine resulting from the SARS-CoV2 pandemic, employers have been particularly interested in virtual primary care as a novel means of expanding primary care services. The purpose of this study is to describe a model of integrated care delivered both in-person and virtually at employer-sponsored health centers nationwide. The key outcomes of this analysis were the proportion of all care delivered in-person and virtually by clinical discipline, the types of care and member satisfaction for care delivered in-person and virtually, and a description of the use of multiple clinical disciplines by the employee population.

**Methods:** Retrospective observational study comparing health services utilization of primary care, behavioral health, and physical medicine services both in-person and virtually in employer-sponsored clinics between January 1, 2020 and June 30, 2021.

**Results:** Of the 331,967 visits with employer-sponsored health center staff, 63% were in-person and 37% were delivered virtually. Most visits were for primary care services (59.5%), with physical medicine visits and behavioral health visits accounting for 25.1% and 15.4%, respectively. Whereas the preponderance of behavioral health visits were virtual visits (72.5%), less than a quarter (18.2%) of physical medicine visits were delivered virtually. 19.6% of patients were seen by more than two clinical disciplines and 2.6% were seen by three different disciplines. Overall, patients were highly likely to recommend the health center across both modalities (Net Promoter Score 89.1 for in-person care and 88.4 for virtual care).

**Discussion:** The future of employer-sponsored integrated team-based care may require a hybrid approach that can lean heavily on virtual visits but requires the infrastructure necessary for in-person care.

## Background

Health care costs in the United States are skyrocketing, with an increase of 5.3% expected for commercial plans in 2021.^1^ In response, self-insured employers, who provide 49% of health care coverage,^2^ have implemented numerous interventions to reduce health care spending while providing high-quality care for their populations. These include on-site/near-site clinics,^3,4^ centers of excellence, direct contracting,^5^ reference pricing,^6^ and condition management programs.^1^ Many of these efforts are aimed at expanding access to primary care (e.g., on-site/near-site clinics), enabling employees to obtain preventive care services and evidence-based condition management, while decreasing downstream high-cost specialists visits (e.g., centers of excellence, narrow networks, and direct contracting).

Since the explosion of telemedicine resulting from the SARS-CoV2 pandemic, employers have been particularly interested in virtual primary care as a novel means of expanding primary care services.^1^ Spurred by the Centers for Medicare and Medicaid Services' decision to ease restrictions on telemedicine, primary care, and specialist care providers across the United States transitioned their practices from in-person to virtual in early March 2020, contributing to widespread adoption of telemedicine services^7^—primary care providers who previously delivered most care in-person offered virtual visits, and virtual care providers who historically delivered tele-urgent care (e.g., Teladoc, AmWell, and Doctor on Demand) offered virtual primary care services. However, several challenges have emerged for both patients and providers as a result of transitioning from the makeshift telehealth solutions rapidly implemented during the pandemic to sustainable virtual primary care. They include social and economic factors (e.g., access to internet connectivity, webcam/video chat, and knowledge of the internet), security breaches, acquiring patients and developing longitudinal relationships with them, coordinating care, managing high-quality referrals, and implementing team-based care so that patients' needs can be comprehensively met.^8–12^ Among these imperatives, team-based care is among the most difficult to achieve in virtual settings.^13^

Although several articles have described shifts in utilization of health care services since the declaration of the COVID-10 pandemic in March 2020,^14,15^ they have not evaluated commercially insured populations receiving care in employer-sponsored clinics with value-based payment structures. Moreover, they have not detailed how use of virtual care may have been differentially adopted by various common clinical disciplines.

The purpose of this article is to describe a model of integrated care being delivered both virtually and in-person at employer-sponsored health centers located nationwide. The key outcomes of interest of this analysis were the proportion of all care delivered in-person versus virtually by clinical discipline, the types of care and member satisfaction for care delivered in-person and virtually, and a description of the use of multiple clinical disciplines by the population. In particular, we provide a descriptive analysis of employees journeys through virtual and in-person visits for primary care, behavioral health, and physical medicine services.

## Methods

### Patient population

To evaluate the types of in-person and virtual care delivered through employer-sponsored health center staff, we included all adult patients (age ≥18) cared for by Crossover Health clinicians between January 1, 2020 and June 30, 2021. Crossover Health provides on-site, near-site, and virtual care for employees and family members of self-insured employers.^3,4^

### Analysis

The key outcomes of interest of this analysis were the proportion of all care delivered in-person versus virtually by clinical discipline, the types of care and patient satisfaction for care delivered in-person and virtually, and a description of the use of multiple clinical disciplines by the population. We used de-identified electronic health records for patients receiving care during the study period from which we extracted the date of visit, type of service provided, and principal diagnosis. Furthermore, clinical disciplines were grouped into three broad categories: (1) primary care (including preventive care, chronic care management, and health coaching); (2) physical medicine (including physical therapy, acupuncture, and chiropractic services); and (3) behavioral health (including psychiatry, psychology, and counseling). At the end of each episode of care, patients were asked the single item Net Promoter Score (NPS) question, “How likely is it that you would recommend the health center to a friend or colleague?.”^16^ NPS for the population are reported on a scale of −100 to +100. We summarized the proportion of visits conducted in-person and virtually and patient satisfaction scores using univariate statistics and report means and standard deviations (SD), unless otherwise noted. We conducted all statistical analyses in Excel 16.5. Given that all data are routinely collected for ongoing patient care, this protocol was considered IRB exempt.

### Intervention

Crossover Health provides integrated in-person and virtual care from employer-based on-site and near-site clinics. The goals of its virtual offering are to preserve the dynamics of its in-person, team-based, integrated care model, as described previously,^3,4^ while including telehealth components to improve access and convenience for members. The hybrid approach of in-person and virtual care between patients and providers accomodates populations that are geographically distributed and provides more flexibility for members, who may choose to visit a physical clinic or receive care remotely.

**FIG. 1.**
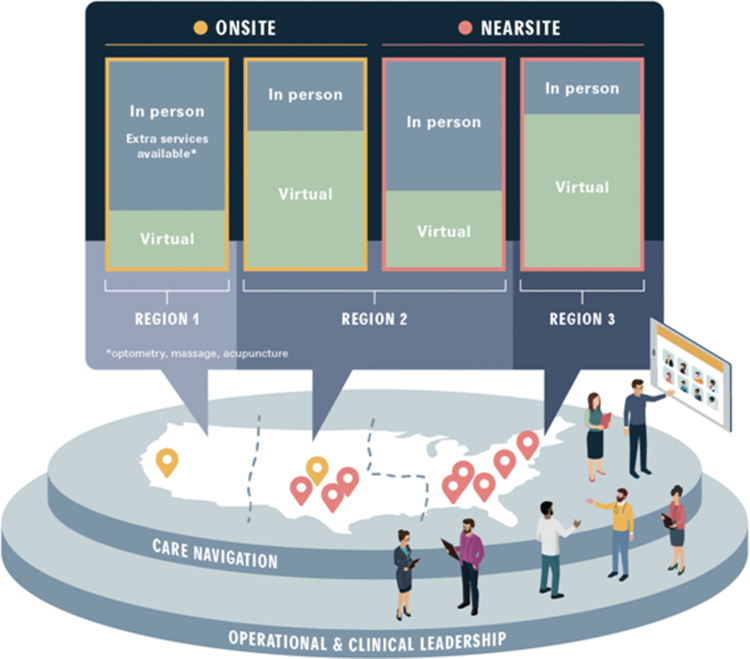
Integrated in-person and virtual care system. This schematic highlights the concepts that the service of geographically distributed employee populations requires combinations of on-site and near-site physical locations with access to virtual care. On-site clinics (represented in *yellow*) typically serve only the employee population while near-site clinics (represented in *red*) can serve both employees and their dependents. Depending on the population, more care might be delivered in-person (*gray*) rather than virtually (*green*). In addition, some services, such as care navigation/referral management, are best managed regionally (i.e., so that care navigators develop expertise in the highest quality providers in their region), whereas other shared services such as operational and clinical leadership are best managed across the entire organization.

The hybrid care model includes six key components that remove barriers to care while improving collaboration and care coordination among providers: team composition, episodes of care, referral management, technology, physical space, and fee structure. Given varied interpretations of what constitutes team-based care^17^—including team composition, processes, and supporting technologies—what follows is a brief description of each key component that facilitates virtual team-based primary care and referral management. Figure 1 graphically represents how the organization operates.

#### Team composition

Teams consist of a mix of clinical disciplines (primary care, behavioral health, physical medicine, and health coaching) that provide integrated multidisciplinary care. Clinical disciplines include multiple provider types: Primary care includes physicians and registered nurse practitioners; behavioral health includes psychologists, social workers, and psychiatrists; physical medicine includes chiropractors, physical therapists, and acupuncturists; health coaching includes licensed coaches and dieticians. Additional team members include optometrists, massage therapists, nurses, and care navigators. Instead of a top-down approach, where primary care providers “quarterback” patient care, each member of the care team works in concert with colleagues to deliver multidisciplinary holistic care to shared patients. Patients may be referred within clinical disciplines (e.g., from a physical therapist to a chiropractor), or they may be referred between disciplines (e.g., from a primary care provider to a psychologist). Patients may also self-refer to any provider.

Care navigators manage referrals to specialists in the community. They develop regional expertise about high quality, lower cost, commonly used providers (e.g., imaging centers and dermatologists), are facile in the use of navigation tools to identify high-quality providers outside the geographic region of a clinic, identify local resources to address care barriers related to social determinants of health, and help patients navigate their employer benefits.

Recruiting, training, and quality assessments are critical in a virtual care environment. Clinicians who excel at delivering in-person care do not necessarily deliver equally high-quality care virtually or enjoy doing so. Clinicians require training on how to use technology efficiently to create a safe empathic environment for the clinical encounter, perform physical examinations virtually, and move seamlessly between synchronous and asynchronous patient interactions.

Virtual processes can replace in-person ones to facilitate team-based care: The care team begins their day with a virtual Zoom “huddle” during which they review the schedule for the day and discuss complex cases. In addition, each clinical discipline (e.g., primary care, physical medicine, and behavioral health) has its own weekly Zoom meeting to discuss specialist-specific issues (e.g., best practices for doing virtual physical therapy examinations). In addition, all members of a care team have “MatterMost” a (Slack-like) Health Insurance Portability and Accountability Act-compliant channel open all day on their workstations to quickly get curbside consults from colleagues.

#### Episodes of care

Providers are trained to consider patient needs in terms of episodes of care.^18^ Episodes include all the services required to manage a condition. Acute issues (e.g., pneumonia, ankle sprain) are discrete episodes that may be resolved (“closed”) but can be reopened if there is a recurrence. Chronic conditions are open episodes that never close. When considering the bundle of services required for any episode, they can be broken down into the evidence-based tasks that can be assigned to various members of the care team. For example, for a patient with pneumonia, the standard episode of care plan may include the following tasks: the primary care provider requests the referral management team to schedule the patient for a chest X-ray, reviews the chest X-ray results, communicates these results to the patient, orders an antibiotic, and schedules a nursing follow-up call. Each of these tasks can be assigned to the relevant members of the care team with “due dates” that can be used to create task lists for care team members. Clinical quality and cost-effectiveness can be evaluated based on task and episode closure rates.

#### Referral management

Many acute and chronic episodes of care require referrals for laboratories, imaging, and specialist visits. Historically, the ordering clinician specified the provider to whom the patient was being referred. However, this requires knowledge of high-quality in-network providers across a range of specialties and geographies, which is impossible for any individual clinician. Instead, centralizing this function with a team of dedicated care navigators can standardize processes and increase referral efficiency (Fig. 1). Specifically, care navigators adhere to standard processes for provider selection, scheduling, document management, and outcomes reporting back to the care team.

#### Technology

Technology is essential for enabling virtual integrated team-based care. The care team and patients have access to the same electronic health record with different views depending upon who is using the system. Patients interact with their care team through an app, which they use to self-refer to any provider, message providers, schedule appointments, and monitor laboratory and other test results. Patients can access the full details for each episode of care, including diagnosis, treatment plan, relevant educational materials, and communication threads between themselves and their care team. Patients may be pushed a set of questions relevant to either an ongoing episode or an annual health risk assessment by providers.

Providers use the app to monitor patient progress, to meet with patients virtually, and to deliver patient resources, including educational materials and treatment information. All members of the care team, including clinicians and care navigators, can see open and closed episodes of care and the tasks assigned to them. Care team members also use other technology specific for their role (e.g., care navigators use navigation tools; physical medicine care providers use MedBridge for prescribing physical therapy; behavioral health providers use Tridiuum for capturing responses related to depression, anxiety, and other common symptoms).

Clinical and operational leaders manage the care across regions and specialties through a set of dashboards with common metrics for all specialties (e.g., caseloads, referral rates, patient satisfaction, NPS, and average episode of care duration) and specialist-specific metrics (e.g., Behavioral Health Index^19^ for behavioral health and Health Confidence Score^20^ for health coaching). Condition-specific programs have their own dashboards (e.g., diabetes program metrics include patient completion of the program, patient satisfaction, change in weight, change in HbA1c, pneumococcal vaccination rate, and percentage of population on a statin).

#### Physical space

Even in a primarily virtual practice, there are several factors that necessitate in-person care, including patients requiring a specific procedure (e.g., phlebotomy), therapeutic intervention (e.g., immunizations), challenging or sensitive physical examination, or member preference. The layout of the clinics to facilitate team-based integrated care has been described previously.^3^ In support of virtual care, new clinics are smaller and designed specifically to deliver care that cannot be done virtually.

#### Fee structure

Employers pay a per member per month fee for employees' and their dependents' access to comprehensive primary care services. This payment model incentivizes provider performance based on outcomes as opposed to quantity of visits, as in traditional fee-for-service arrangements. The amount that patients pay for care depends on their employer-sponsored benefits; however, the majority have a copay that is similar to or less expensive than they would pay for care in the community. These copays range from $0 to $90 and are the same across all clinical disciplines and are the same for virtual and in-person visits. Providers are salaried and have no incentives to see patients multiple times or through a particular modality (virtually or in-person).

## Results

### Demographics

Table 1 presents demographic characteristics of the 67,478 patients seen by Crossover Health staff between January 1, 2020 and June 30, 2021: 54% were male with a mean age of 38.1 years (SD 11.0).

**Table 1.** Patient Characteristics

Characteristic	Mean or % (SD)
Age (years)	38.1 (11)
Gender	
% Male	53.9
% Female	46.0
% Other	0.1
Average number of visits per patients	
Total pre-COVID	5.6 (4.4)
Total post-COVID	4.6 (5.3)
Primary care pre-COVID	3.3 (2.7)
Primary care post-COVID	3.1 (3.3)
Behavioral health pre-COVID	5.9 (7.3)
Behavioral health care post-COVID	7.9 (9.3)
Physical medicine pre-COVID	6.8 (6.5)
Physical medicine post-COVID	5.2 (8.1)

SD, standard deviation.

### Team-based care

**FIG. 2.**
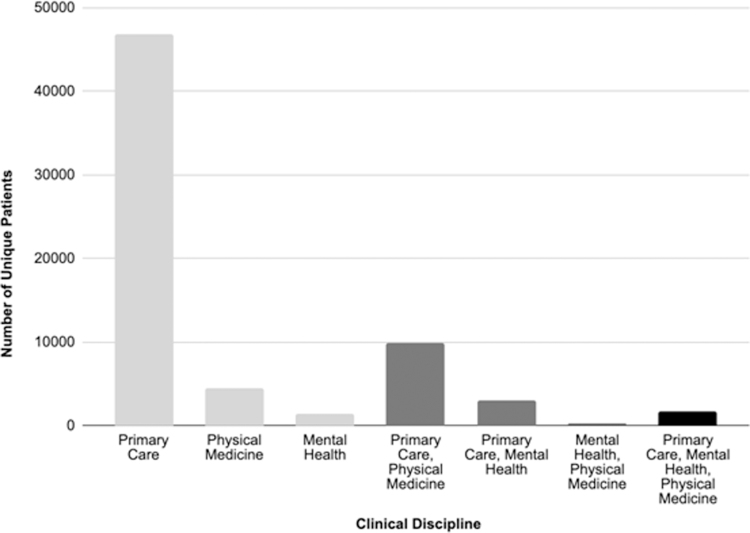
Distribution of care by multiple members of the integrated clinical team. The number of patients seen by a single clinical discipline are denoted in *light gray*, seen by two clinical disciplines are denoted in *dark gray*, and seen by three clinical disciples in *black*.

Most visits during the study interval were for primary care (59.5%), with physical medicine visits and behavioral health visits accounting for 25.1% and 15.4%, respectively. However, 19.6% of patients were seen by two clinical disciplines and 2.6% were seen by three different disciplines (Fig. 2). An example of team-based care includes that all patients seen for an initial in-person visit with a physical medicine provider have their blood pressure checked. This resulted in 39% of members with a blood pressure screen being referred to primary care and 20% of all members with a blood pressure screen in physical medicine receiving a new diagnosis of hypertension.

### In-person versus virtual visits

**FIG. 3.**
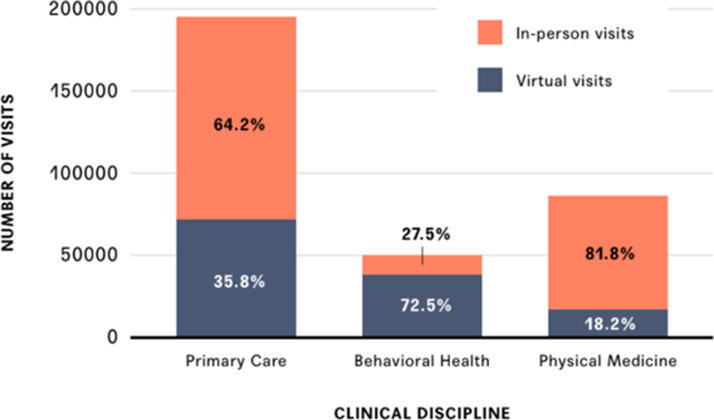
Frequency of in-person and virtual visits by clinical discipline. In-person visits are denoted in *orange* and virtual visits are denoted in *blue* for each clinical discipline.

**FIG. 4.**
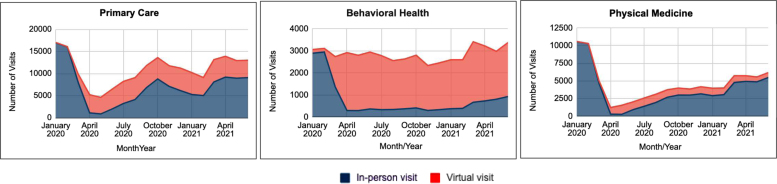
In-person and virtual visits over the study period by clinical discipline. In-person visits are denoted in *blue* and virtual visits are denoted in *orange* for each clinical discipline over the study period.

Of the 331,967 visits completed during the study period, 63% were in-person and 37% were delivered virtually (Fig. 3). On average, patients had 5.6 (SD 4.4) total visits pre-COVID and 4.6 (SD 5.3) visits after the start of the pandemic (Table 1). Figure 4 shows that in-person visits dominated prepandemic (in January and February of 2020), accounting for 99.2% of all visits. The total number of in-person visits fell precipitously to 18% of baseline monthly visits; however, virtual visits increased considerably to 82% of all visits. By April 2020, the number of virtual visits plateaued, averaging 7919 (SD 571) visits per month.

#### Primary care

Over the study interval, virtual visits accounted for 35.8% of all primary care visits. These virtual visits did not offset the decline in in-person care (in January 2020 there were 17,045 primary care visits compared with 13,009 in June 2021; Fig. 4). Compared with prepandemic, the number of primary care visits per episode of care decreased by 6% after March 2020. The average time between visits increased for primary care from 47.4 to 99.6 days. Physical examinations delivered both in person and virtually accounted for the preponderance (16.6%) of all visits (Table 2). Notably, 5.4% of all visits were physical examinations delivered virtually. Immunizations (which cannot be delivered virtually) were a key reason for in-person primary care visits. COVID-related care was not in the top 5 diagnoses for primary care visits but was in the top 10.

**Table 2.** Top Five Diagnosis for In-Person and Virtual Visits

In-person visit		Virtual visit	
Primary care			
**General adult medical examination**	**11.2%**	**Dietary counseling**	**7.6%**
Immunization	3.0%	**General adult medical examination**	**5.4%**
**Hypertension**	**2.9%**	Anxiety disorder	2.6%
**Dietary counseling**	**2.7%**	**Hypertension**	**2.3%**
Hyperlipidemia	1.7%	Major depressive disorder	1.8%
Behavioral health			
**Anxiety disorder**	**1.9%**	**Anxiety disorder**	**9.8%**
**Problems in relationship with spouse or partner**	**0.9%**	**Major depressive disorder**	**3.3%**
**Major depressive disorder**	**0.7%**	**Adjustment disorder with mixed anxiety and depressed mood**	**2.7%**
**Adjustment disorder with mixed anxiety and depressed mood**	**0.6%**	**Problems in relationship with spouse or partner**	**2.0%**
Physical medicine			
**Low back pain**	**11.0%**	**Low back pain**	**2.0%**
**Cervicalgia**	**12.6%**	**Cervicalgia**	**1.4%**
Pain in thoracic spine	**7.8%**	Knee pain	**2.7%**

Diagnoses in bold are in the top five conditions treated both in-person and virtually.

#### Behavioral health

Over the study interval, the preponderance of behavioral visits were virtual visits (72.5%). Virtual visits more than offset declines in in-person care given that the number of behavioral health visits increased from 3056 in January 2020 to 3373 in June 2021 (Fig. 4). Also, the number of visits per episode of care increased by 34%. The average time between visits increased from 19.7 to 23.8 days. Behavioral health diagnoses, including depression, anxiety, adjustment disorder, and relationship problems with a spouse or partner, accounted for 26.3% of the top diagnoses both among behavioral health visits and primary care visits.

Among those patients with behavioral health (BH) conditions who received in-person care pre-COVID, 24.8% went on to receive subsequent virtual BH care, whereas 14% received no additional BH care. 43.5% patients were newly diagnosed with BH after the onset of the pandemic.

#### Physical medicine

Over the study interval only 18.2% of physical medicine visits were delivered virtually. Compared with prepandemic, the total number of physical medicine visits decreased significantly from 10,575 in January 2020 to 6175 in June 2021 (Fig. 4). Also, the number physical medicine visits per episode of care decreased by 23.5%. The average time between each visit increased for physical medicine from 17.6 to 44.4 days. Much of the care delivered by physical medicine practitioners after the start of the pandemic was related to injuries or discomfort associated with change in either ergonomics (i.e., poor workstation setup at home compared with what they used in their workplace) or preferred exercise (e.g., started running rather than going to the gym). Notably, the increase in in-person physical medicine visits in February 2021 was associated with increased numbers of employees returning to the workplace.

**FIG. 5.**
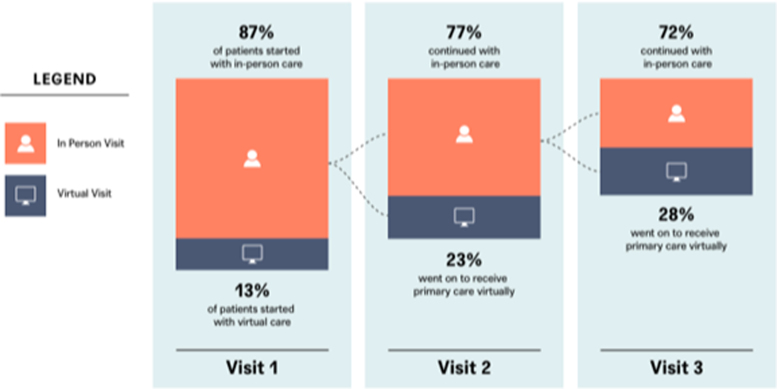
Patient flows through in-person and virtual care. This schematic describes the flow of patients through in-person and virtual care during the study interval. The most prevalent path was for those people who started with an in-person visit and went on to have multiple additional in-person visits. In-person visits are denoted in *orange* and virtual visits are denoted in *blue* for each clinical discipline.

Figure 5 depicts the flow of patients based on where they had their first visit during the study period, moving through virtual and in-person care. The majority of patients (87.1%) started their care journeys with an in-person visit. Of those who required a second visit care, 77.4% went on to continue to receive only in-person care and 22.6% went on to receive some of their care virtually. Of those who continued with a third visit, 71.6% received in-person care and 28.4% received follow-up care virtually.

Overall, patients were highly likely to recommend the health center across both modalities (NPS 89.1 for in-person care and 88.4 for virtual care).

## Discussion

As a result of the SARS-CoV2 pandemic, telehealth services have now become part of the expected offering for commercially insured populations. This analysis of integrated team-based care delivered both in-person and virtually has six key findings. First, although virtual care is particularly valuable for patients with behavioral health conditions, patients seeking primary care and physical medicine services require both in-person and virtual care. This implies that neither virtual-only nor in-person only primary care solutions can meet all the needs of an employee population. Our findings corroborate those of others that demonstrate both that behavioral health issues dominate both primary care and behavioral health visits^21,22^ and that the care of people with behavioral health issues is well accommodated in the virtual setting.^23,24^ Given that others have demonstrated lower utilization of telehealth services to offset deferred in-person care during 2020 among low-income and non-white populations,^14^ evaluating how these social determinants might have affected health care utilization among patients receiving care from employer-sponsored health centers would be an important extension of this study.

Second, by the end of June 2021, physical medicine services had not yet returned to their prepandemic levels, although monthly visits were on the rise. Other published studies have shown faster returns to baseline health services delivery generally but have not specifically evaluated physical medicine services.^14,15^ We speculate that employees who were working from home did not consider going back to the workplace just to access care at employer-sponsored on-site and near-site clinics. Moreover, employees in need of physical medicine services, which are often considered to require in-person interactions with both clinicians and equipment, may not have been aware of virtual physical medicine options for care coming from their on-site clinic providers.

Third, the diagnoses associated with in-person and virtual visits are very similar, suggesting that members can be seen virtually for a wide spectrum of conditions including physical examinations. A priority for future research includes an analysis of the patient factors or clinical situations that necessitate in-person care.

Fourth, the frequency of patients seeing multiple professionals underscores the importance of team-based care that can be delivered both in-person and virtually. Moreover, having an integrated approach that includes primary care, physical medicine, behavioral health, and coaching enables more widespread screening for disease, regardless of the team with whom the patient initiates their care journey. This was evident in the finding that one in five patients being seen in physical medicine were found to have a new diagnosis hypertension that was immediately addressed in primary care (significantly higher than what has been reported in the literature of community-based physical therapy).^25,26^ In addition, 10.4% of patients seen in primary care were diagnosed with depression or anxiety for which they were seen by a behavioral health clinician. These findings underscore the importance of providing integrated team-based care for employee populations.

Fifth, this article describes the elements of the offering that facilitate this team-based approach including team composition, integrating the concept of episodes of care, referral management, shared technology, physical space, and fee structure. Shifting from an in-person to virtual offering increases the burden of technology use on providers.^27^ As the infrastructure supporting virtual team-based care improves, a key area for innovation will be to address the burden on providers to use numerous tools for various aspects of patient care (e.g., tools for intra-team and team–patient communication; electronic health records; tools for prescribing medications and physical therapy; tools for outcomes assessment). In addition, an episode of care approach requires team alignment when it comes to managing care tasks: understanding what constitutes an episode, knowing what processes are needed to deliver appropriate health care services at the right time, and sharing accountability across a range of providers and settings for both outcomes and costs.^18,28^ An episodes-based approach necessitates technology that can capture and integrate data that enable this level of care coordination, so that quality of care does not get lost in a handoff from one provider to another.^18^ Future research should evaluate the extent to which episode-of-care approaches increase or reduce missed or duplicative health care services when teams fail to coordinate effectively.

Finally, member satisfaction with in-person and virtual care was excellent (NPS 89.1 for in-person care and 88.4 for virtual care) and compares favorably with other health care organizations [e.g., CVS Health (−10), Cigna (−1), and Walgreens (25)].^16^ This suggests that patient preference for modality of care should be solicited as part of a patient-centric approach to primary care. Among the factors associated with patient satisfaction with telehealth, including improved patient–provider communication, ease of access, and low costs, patients identify improved self-efficacy as a key benefit of virtual care.^29,30^ In the employer-sponsored health centers, staff had no incentives for providing care in-person or virtually, so the differences seen in this study were largely driven by patient choice. The implications of this finding suggest that virtual only offerings for employee populations may not adequately meet the needs of the preponderance of the population.

This analysis had four key limitations: First, the results are limited to commercially insured populations and do not extend to Medicare, Medicaid, or uninsured populations. Second, the number of interdepartmental referrals (e.g., from primary care to behavioral health) were underreported given that they did not include an assessment of frequent curbside consults. Third, the differences in the quality of clinical care delivered between in-person and virtual offering were not investigated. Given that care was delivered through both modalities by the same clinicians using the same organizational protocols, we anticipate that care would be similar but this is a key target for future evaluations. Finally, provider satisfaction was not measured and may vary considerably for clinicians providing in-person, asynchronous, and synchronous virtual care. Given that provider satisfaction is a critical aspect of the Quadruple Aim of health^31^ and a determinant of sustained delivery of telehealth services, provider training and feedback on how best to incorporate virtual care into clinical practice^32^ is essential for the ongoing development of employer-sponsored care models and may be key to reducing provider burnout.

## Conclusions

High rates of deferred primary care in the wake of COVID-19,^15^ including a decrease in visits related to preventive and elective procedures as well as chronic disease management,^15,33^ and an increase in demand for behavioral health services^34^ all necessitate interprofessional collaboration and coordination to manage the care of employee populations postpandemic. The results of this study suggest that the future of employer-sponsored integrated team-based care may require a hybrid approach that can lean heavily on virtual visits but requires the infrastructure necessary for in-person care.

## Abbreviations Used

NPS: Net Promoter Score
SD: standard deviation
